# Influenza-associated MOG antibody-positive longitudinally extensive transverse myelitis: a case report

**DOI:** 10.1186/s12883-014-0224-x

**Published:** 2014-11-30

**Authors:** Haruka Amano, Nobukazu Miyamoto, Hideki Shimura, Douglas Kazutoshi Sato, Kazuo Fujihara, Shinichi Ueno, Ryota Nakamura, Yuji Ueno, Masao Watanabe, Nobutaka Hattori, Takao Urabe

**Affiliations:** Department of Neurology, Juntendo University Urayasu Hospital, 2-1-1 Tomioka, Urayasu, Chiba 279-0021 Japan; Department of Neurology, Juntendo University School of Medicine, Tokyo, Japan; Department of Neurology, Tohoku University Graduate School of Medicine, Miyagi, Japan

**Keywords:** Myelin-oligodendrocyte glycoprotein antibody, Longitudinal extensive transverse myelitis, Neuromyelitis optica, Aquaporin-4 antibody

## Abstract

**Background:**

Myelin-oligodendrocyte glycoprotein antibody (MOG antibodies) was found in various demyelinated diseases. This is the first report of a patient with longitudinally extensive transverse myelitis with an extremely high titer of MOG antibodies after an influenza infection. This case supports the view that MOG antibodies are linked to longitudinally extensive transverse myelitis and that influenza infection might trigger the MOG antibodies.

**Case presentation:**

A 32-year-old healthy male developed high fever, dysesthesia and paraesthesia below the C2 area, muscle weakness of the bilateral lower extremities, and urinary retention ten days after an influenza type A infection. Magnetic resonance imaging revealed a longitudinal lesion in the spinal cord extending from C2 to the spinal conus. There were no lesions in the brain or optic nerves. Established cell-based immunoassays revealed that he was positive for MOG antibodies (titer = 65,536) and negative for anti-aquaporin 4 antibodies (AQP4 antibodies). He fully recovered after steroid pulse therapy followed by 60 mg prednisolone.

**Conclusion:**

This is the first report of influenza A-associated longitudinally extensive transverse myelitis with a high titer anti-MOG antibodies. Our case report supports a relationship between anti-MOG antibodies and longitudinally extensive transverse myelitis, which was triggered by influenza infection. Further studies are needed to establish the clinical significance of anti-MOG antibodies for diagnosis, treatment, and prognosis.

## Background

Myelin oligodendrocyte glycoprotein (MOG) localizes on the outermost surface of the myelin sheath and oligodendrocytes in the central nervous system (CNS). Autoantibodies against MOG are reportedly found in patients with a spectrum of inflammatory demyelinating diseases of the CNS, including acute disseminated encephalomyelitis, multiple sclerosis, transverse myelitis, and neuromyelitis optica (NMO) [1]. NMO is a severe inflammatory disorder of the central nervous system that predominantly affects the optic nerves and spinal cord. Most patients with NMO have antibodies against the water channel aquaporin-4 (AQP4 antibodies), which are thought to be pathogenic [2-4]. However, some patients are seronegative for AQP4 antibody; the lack of a biomarker makes diagnosis and management of these patients difficult. Many clinicians perceive these patients to be similar to those with AQP4-Abs and manage them in the same way. Recently, some groups showed that some AQP4 antibody seronegative patients have antibodies against MOG [5-7]. They also demonstrated differences in clinical phenotypes and responsiveness of therapy between the AQP4 antibody seronegative and MOG antibody seropositive patients with NMO/NMO spectrum disorder (NMOSD). Detection of MOG antibodies is important for patients management.

Mice that are transgenic for MOG-specific T-cell and B-cell receptors develop spontaneous experimental autoimmune encephalomyelitis (EAE) [8-11]. Recent studies suggest that MOG-related EAE can mimic a neurological syndrome closely resembling NMO. Although the clinical spectrum of MOG IgG associated diseases in humans is reflected in different experimental models, the role of MOG antibodies in pathogenesis is still unclear. Here we describe a patient who suffered from longitudinal extended TM (LETM) with high-titer MOG antibodies following an influenza-A infection and his AQP4 antibody was negative.

## Case presentation

A 32-year-old male, without any relevant medical history, felt general fatigue and had a high fever of 38.9 degrees Celsius. The following day, he was diagnosed with influenza type A by nasal swab test at a clinic, and prescribed oseltamivir. His symptoms were ameliorated sufficiently that he was able to return to work from day 5 and go winter climbing on days 6–7. On day 9, however, he experienced whole body pain, urinary retention, and weakness of the bilateral lower extremities; these symptoms then became exacerbated. Eventually, he was unable to walk by himself, and came to our hospital on day 10. His body temperature was 39.4 degrees Celsius and there was marked bilateral lower extremity weakness. Deep tendon reflexes were normal in both the upper and lower limbs. He had paraesthesia and dysesthesia in the entire area below level C2. Both superficial and deep sensory systems were disturbed in the same area, especially in the lateral sides of the femur (50% decrease) and crus (40% decrease). Meningial signs, such as neck stiffness and Kernig’s sign, were positive. He had no visual field deficits.

**Figure 1:**
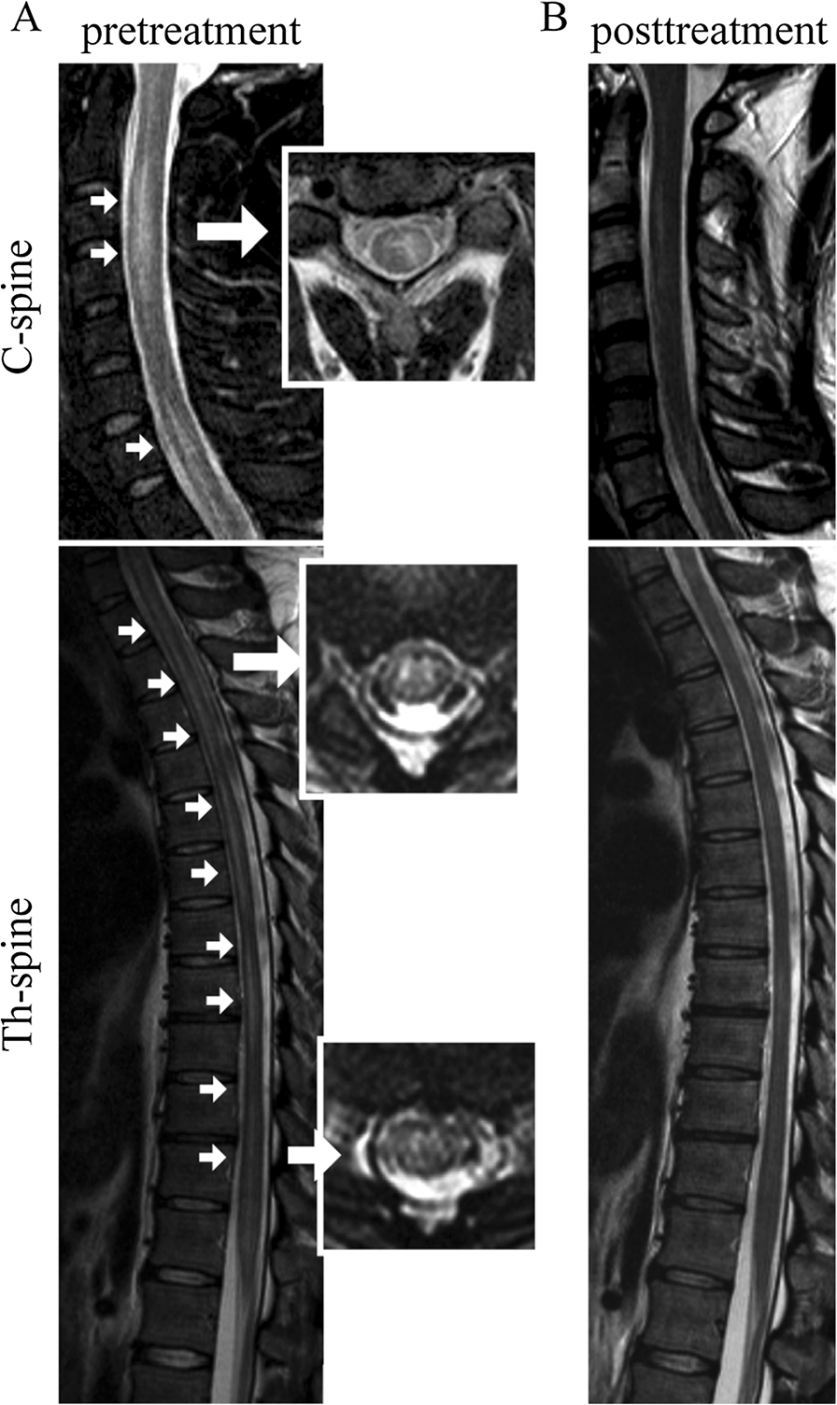
**T2-imaging MRI of cervical (C)/thoracic (Th) spinal cord on admission (A) and after treatment (B).** On admission (day 10), there was a longitudinal T2 high intensity area, extending from C2 to the conus (arrowheads), but the lesion resolved completely after treatment (day 20).

His laboratory data showed a white blood cell count of 13800/μl (Neutrophil 83%) and a c-reactive protein level of 0.4 mg/dl. An anti-nuclear antibody test revealed a titer of 1:320 with a nucleolar pattern, but collagen diseases associated with nucleolar antibodies, such as systemic lupus erythematosus and scleroderma, were negative according to additional tests of other antibodies. The cerebrospinal fluid exhibited a cell count of 247 cells/μl (monocyte 77.1%, lymphocyte 54.5%), an elevated protein level (92 mg/dl), and a myelin basic protein concentration over 2000 pg/ml (IL-6 9490 pg/ml), suggesting strong inflammation of the central nervous system. The oligoclonal band was negative. Established cell-based immunoassays revealed that he was positive for anti-MOG antibodies and negative for anti-AQP4 antibodies. His MOG antibody titer was as high as 65,536, which was extremely high compared to past report in the patients with demyelinated diseases included with NMOSD, which median (range) MOG antibody titers was 4,096 (128–32,768) in single attack patients [12]. T2 imaging MRI showed a long hyperintense lesion in the spinal cord extending from C2 to the medullary conus, mainly lying in thoracic spinal cord. The lesion occupied grey matter, making “H” shape, partly spreading to white matter. The lesion was not enhanced by gadolinium-DTPA (Figure 1). Nerve conduction velocity was normal. We diagnosed him with anti-MOG antibody-positive longitudinally extensive transverse myelitis, and started immunosuppression therapy with intravenous methylprednisolone (1000 mg/day) for three consecutive days, followed by oral prednisolone (60 mg per day). The patient was able to use a wheelchair without any help on day 5 after admission, and he recovered the full strength of his lower extremities by day 6. Paraesthesia and sensory loss gradually improved and resolved by day 10; dysesthesia in the bilateral lower limbs and thermoanesthesia below the Th4 level also improved, and resolved completely one month later. After two weeks of 60 mg per day prednisolone, we reduced it 10 mg a week and finally finished it after 6 weeks. After one week of therapy, the MOG antibody titer declined to 16,384, one fourth of the first laboratory data. After one month, the MOG antibody titer decreased to 4,096.

## Discussion

Here, we describe the first reported case of a patient with LETM following an influenza type A infection who was also positive for anti-MOG autoantibodies. Recently, it was reported that autoantibodies against MOG are found in patients with a spectrum of inflammatory demyelinating diseases of the CNS. Among AQP4 antibody seronegative NMO patients, 21.1% were reported to have high titers of MOG antibodies [6]. MOG antibody positive NMO has specific clinical and epidemiological features different from AQP4 positive NMO [6]. The sexual ratio is 44:56, while 90% of AQP4-positive NMO patients are female. The average age of onset in MOG-positive NMO is 32.29 ± 17.1 years, obviously younger than the 44.86 ± 14.8 years reported for AQP4 antibody positive NMO patients. MOG antibody positive patients had better outcomes from the episode, with better recovery, expanded disability status scale (EDSS) scores, and a low risk for visual and motor disability [6]. On MRI, a thoracic spinal lesion involving the conus and brain lesions adjacent to the fourth ventricle are seen more frequently in MOG antibody positive NMO than in AQP4 antibody positive NMO. High anti-MOG antibody titers are frequently observed among pediatric patients with recurrent optic neuritis [13]. Huppke et al. reported that seven pediatric patients with acute disseminated encephalomyelitis (ADEM) followed by optic neuritis were positive for anti-MOG antibodies, but not anti-AOP4 antibodies [14].

Influenza infection triggers various autoimmune diseases, included neurological disorders (e.g., Guillain-Barre syndrome and ADEM) [15-17]. However, not only influenza infection, influenza vaccination could be a possible cause of CNS demyelinating diseases [18]. Among 71 cases of post-vaccination CNS demyelinating diseases, influenza vaccination was related to 21 cases, occupying the biggest ratio. Majority of demyelination (especially following influenza vaccination) occur in optic nerves and myelon. This predisposition to the spinal cord and the optic nerves is reminiscent to NMO/NMOSD. In general, the symptoms of post-vaccination demyelinating diseases appear 14.2 days (mean) after immunization [18]. Here, we present the first report of a patient with anti-MOG antibody-positive LETM after influenza infection. Previously, Nakamura et al. reported a patient with NMO who showed optic neuritis and longitudinally extensive lesions in the thoracic cord without anti-AQP4 antibodies. They did not examine the titer of anti-MOG antibodies [19]. The mechanisms of development and function of anti-MOG antibodies remain unclear. Our case showed that influenza infection may trigger anti-MOG-IgG positive myelitis; following treatment, the titer of anti-MOG antibody decreased together with clinical symptoms. Additional studies are needed to establish the clinical significance of anti-MOG antibodies for diagnosis, treatment, and prognosis.

## Conclusion

We have presented the first report of a patient with MOG-positive LETM at the C2 to conus level without optic neuritis after influenza A infection.

## Consent

Written informed consent was obtained from the patient for publication of this Case report and any accompanying images. A copy of the written consent is available for review by the Editor of this journal.

## References

[1] Reindl M, Di Pauli F, Rostasy K, Berger T (2013). The spectrum of MOG autoantibody-associated demyelinating diseases. Nat Rev Neurol.

[2] Lennon VA, Kryzer TJ, Pittock SJ, Verkman AS, Hinson SR (2005). IgG marker of optic-spinal multiple sclerosis binds to the aquaporin-4 water channel. J Exp Med.

[3] Saadoun S, Waters P, Bell BA, Vincent A, Verkman AS, Papadopoulos MC (2010). Intra-cerebral injection of neuromyelitis optica immunoglobulin G and human complement produces neuromyelitis optica lesions in mice. Brain.

[4] Ratelade J, Zhang H, Saadoun S, Bennett JL, Papadopoulos MC, Verkman AS (2012). Neuromyelitis optica IgG and natural killer cells produce NMO lesions in mice without myelin loss. Acta Neuropathol.

[5] Mader S, Gredler V, Schanda K, Rostasy K, Dujmovic I, Pfaller K, Lutterotti A, Jarius S, Di Pauli F, Kuenz B, Ehling R, Hegen H, Deisenhammer F, Aboul-Enein F, Storch MK, Koson P, Drulovic J, Kristoferitsch W, Berger T, Reindl M (2011). Complement activating antibodies to myelin oligodendrocyte glycoprotein in neuromyelitis optica and related disorders. J Neuroinflammation.

[6] Kitley J, Waters P, Woodhall M, Leite MI, Murchison A, George J, Kuker W, Chandratre S, Vincent A, Palace J (2014). Neuromyelitis optica spectrum disorders with aquaporin-4 and myelin-oligodendrocyte glycoprotein antibodies: a comparative study. JAMA Neurol.

[7] Rostasy K, Mader S, Hennes EM, Schanda K, Gredler V, Guenther A, Blaschek A, Korenke C, Pritsch M, Pohl D, Maier O, Kuchukhidze G, Brunner-Krainz M, Berger T, Reindl M (2013). Persisting myelin oligodendrocyte glycoprotein antibodies in aquaporin-4 antibody negative pediatric neuromyelitis optica. Mult Scler.

[8] Bettelli E, Pagany M, Weiner HL, Linington C, Sobel RA, Kuchroo VK (2003). Myelin oligodendrocyte glycoprotein-specific T cell receptor transgenic mice develop spontaneous autoimmune optic neuritis. J Exp Med.

[9] Bettelli E, Baeten D, Jager A, Sobel RA, Kuchroo VK (2006). Myelin oligodendrocyte glycoprotein-specific T and B cells cooperate to induce a Devic-like disease in mice. J Clin Invest.

[10] Krishnamoorthy G, Lassmann H, Wekerle H, Holz A (2006). Spontaneous opticospinal encephalomyelitis in a double-transgenic mouse model of autoimmune T cell/B cell cooperation. J Clin Invest.

[11] Berer K, Mues M, Koutrolos M, Rasbi ZA, Boziki M, Johner C, Wekerle H, Krishnamoorthy G (2011). Commensal microbiota and myelin autoantigen cooperate to trigger autoimmune demyelination. Nature.

[12] Sato DK, Callegaro D, Lana-Peixoto MA, Waters PJ, de Haidar Jorge FM, Takahashi T, Nakashima I, Apostolos-Pereira SL, Talim N, Simm RF, Lino AM, Misu T, Leite MI, Aoki M, Fujihara K (2014). Distinction between MOG antibody-positive and AQP4 antibody-positive NMO spectrum disorders. Neurology.

[13] Rostasy K, Mader S, Schanda K, Huppke P, Gartner J, Kraus V, Karenfort M, Tibussek D, Blaschek A, Bajer-Kornek B, Leitz S, Schimmel M, Di Pauli F, Berger T, Reindl M (2012). Anti-myelin oligodendrocyte glycoprotein antibodies in pediatric patients with optic neuritis. Arch Neurol.

[14] Huppke P, Rostasy K, Karenfort M, Huppke B, Seidl R, Leiz S, Reindl M, Gartner J (2013). Acute disseminated encephalomyelitis followed by recurrent or monophasic optic neuritis in pediatric patients. Mult Scler.

[15] Mizuguchi M (2013). Influenza encephalopathy and related neuropsychiatric syndromes. Influenza Other Respir Viruses.

[16] Ekstrand JJ (2012). Neurologic complications of influenza. Semin Pediatr Neurol.

[17] Akins PT, Belko J, Uyeki TM, Axelrod Y, Lee KK, Silverthorn J (2010). H1N1 encephalitis with malignant edema and review of neurologic complications from influenza. Neurocrit Care.

[18] Karussis D, Petrou P (2014). The spectrum of post-vaccination inflammatory CNS demyelinating syndromes. Autoimmun Rev.

[19] Nakamura Y, Ikeda K, Yoshii Y, Ito H, Hirayama T, Kawabe K, Kano O, Iwasaki Y (2011). Influenza-associated monophasic neuromyelitis optica. Intern Med.

